# Organic Zinc and Selenium Supplementation of Late Lactation Dairy Cows: Effects on Milk and Serum Minerals Bioavailability, Animal Health and Milk Quality

**DOI:** 10.3390/ani15040499

**Published:** 2025-02-10

**Authors:** Gabriel S. Klein, Karoline W. Leal, Camila A. Rodrigues, Taynara M. R. Draszevski, Andrei L. R. Brunetto, Maksuel G. Vitt, Mathias S. Klein, Vitoria H. Cauduro, Erico M. M. Flores, Gilnei B. da Silva, Margarete D. Bagatini, Alana B. de M. Chitolina, Matheus D. Baldissera, Aleksandro S. Da Silva

**Affiliations:** 1 Department of Animal Science, Universidade do Estado de Santa Catarina, Chapecó 89815-630, Brazil; gabriel.klein@edu.udesc.br (G.S.K.); ca.rodrigues@edu.udesc.br (C.A.R.); taynarareginato25@gmail.com (T.M.R.D.); 2 Graduate Program in Veterinary Medicine, Universidade Federal de Santa Maria, Santa Maria 97105-900, Brazil; karolwagnerleal@gmail.com; 3 Graduate Program in Animal Science, Universidade do Estado de Santa Catarina, Chapecó 89815-630, Brazil; andreibrunetto03@gmail.com (A.L.R.B.); mak-witt@hotmail.com (M.G.V.); 4 Graduate Course of Specialization in Plant Production Systems, Instituto Federal de Educação, Ciência e Tecnologia do Rio Grande do Sul, Sertão 99170-000, Brazil; mathias.klein@hotmail.com; 5 Graduate Program in Chemistry, Department of Chemistry, Universidade Federal de Santa Maria, Santa Maria 97105-900, Brazil; 6 Department of Chemistry, Universidade Federal de Santa Maria, Santa Maria 97105-900, Brazil; ericommf@gmail.com; 7 Multicentric Postgraduate Program in Biochemistry and Molecular Biology, Universidade do Estado de Santa Catarina, Lages 88520-000, Brazil; gilneibrunosilva@gmail.com; 8 Graduate Program in Biomedical Sciences, Universidade Federal da Fronteira Sul, Chapecó 89815-899, Brazil; margarete.bagatini@uffs.edu.br; 9 Undergraduate in Biomedicine, Universidade Franciscana, Santa Maria 97010-032, Brazil; alana.chitolina@ufn.gov.br; 10 Laboratory of Bioprospecting and Experimental Biology, Universidade Franciscana, Santa Maria 97010-032, Brazil; matheus.dellamea@ufn.edu.br

**Keywords:** immunity, inflammation, microminerals, milk fat, nutraceutical, supra-nutritional

## Abstract

This study determined whether organic zinc and selenium supplementation of late lactation dairy cows positively affects immunity, oxidative status, milk quality (especially mineral levels), biochemical and hematologic parameters, and production efficiency. Twenty Jersey cows were divided into three groups: Control (n = 6)—without organic supplementation; Zinc (n = 7)—zinc supplementation (zinc amino acid chelate) and Selenium (n = 7)—selenium supplementation (selenium amino acid complex). The basal diet contained inorganic minerals. Blood and milk samples were collected on days 1, 14 and 28. Serum selenium concentration was higher in the Selenium group, and zinc level in milk was higher in the Zinc group. On day 28, supplementations resulted in higher lymphocyte counts, and lower serum creatine kinase, myeloperoxidase activity, levels of reactive oxygen species, thiobarbituric acid-reactive substances, and iron. In milk, lower somatic cell count was also observed when cows were zinc or selenium supplemented compared to the control. Lower serum cholinesterase activity and higher heavy chain immunoglobulin concentration were observed on days 14 and 28. Selenium supplementation resulted in a higher immunoglobulin A concentration on days 14 and 28, and lower ceruloplasmin concentration on day 28 compared to Control, as well as a lower haptoglobin concentration on day 28. The Selenium group also had lower milk fat content compared to the Control. Supplementations changed the milk fatty acid profile, producing a higher unsaturated fatty acid/saturated fatty acid ratio. There was no effect on lactation persistence. It is concluded that mineral supplementation with selenium and zinc benefits immune, antioxidant, and anti-inflammatory responses. Conversely, milk quality was affected both positively and negatively.

## Simple Summary

Supplementation of organic zinc and selenium in late lactation dairy cows, in the form of chelated zinc amino acid and selenium amino acid complex, had positive effects on immunity and antioxidant activity, as well as on biochemical parameters linked to inflammatory responses. Feed efficiency positively responded to supplementation. The zinc concentration in the group’s milk supplemented with organic zinc was higher, indicating greater mineral bioavailability. Despite the change in the fatty acid profile that may benefit human health and the reduction, with selenium supplementation alone, of urea nitrogen content, solids content showed a tendency to decrease, and selenium supplementation induced a reduction in fat percentage.

## 1. Introduction

Lactating dairy cows are constantly challenged due to high physiological stress [1], and this constant state of challenge, which is the high milk production associated with the physiological stress of lactation, can increase susceptibility to metabolic and sanitary disorders, which harm the animal’s health and can reduce milk production and quality [2,3]. In addition, nutritional requirements, such as energy and protein, are substantially increased in the final third of lactation as pregnancy advances [4,5], highlighting the cost that pregnancy represents for the cow. The approach of peripartum demands greater attention to the animal since this period is related to the increased incidence and severity of diseases and immunological and inflammatory dysfunction, of which oxidative stress is one of the causes [6]. Therefore, it is of fundamental importance to maintain a good state of health of the cow to minimize the negative impacts associated with the arrival of the peripartum period. The increase in health can be determined by improving antioxidant activity and immunological status, which also contribute to milk quality [7]. Recognition of the importance of immunity for mammary and uterine health is growing [8], and, with this, the cow, the fetus, and milk consumers can benefit.

One possibility that can be used to improve health is the supplementation of microminerals in organic forms, especially those associated with immune and antioxidant responses, such as zinc and selenium. Zinc is involved in several aspects of the immune system and is a component of the antioxidant system [9,10,11]. Selenium is a component of specific proteins, selenoproteins, which have several functions, including acting as antioxidants and involvement in immune and inflammatory responses [9,12,13,14]. The forms usually used in supplementation are inorganic, zinc oxide, sulfate [15,16], and sodium selenite [17]. However, the absorption efficiency of these forms is low, with the zinc absorption coefficient being 0.16 for oxide and 0.20 for sulfate [9]. The absorption of selenium from inorganic sources occurs by passive diffusion, with little retention of the mineral and a large proportion excreted [18]. On the other hand, microminerals in organic form, chelated or complexed with organic compounds or molecules, such as amino acids [19], when adequately chelated, have better bioavailability; that is, they are better absorbed and utilized [20]. However, there may be discrepancies in the results when using different organic minerals since the chemical form affects the absorption and use of microminerals [21].

It is well known that the use of organic forms such as zinc amino acid chelate and zinc proteinate chelate with strong chelation strength was able to increase the humoral immune response of lactating cows [22]. In addition, prepartum supplementation of zinc methionine can improve antioxidant activity [23]. Additional supplementation of zinc methionine during lactation, in addition to the inorganic source, reduces the somatic cell count in milk, with the magnitude of reduction increasing with higher levels of supplemented zinc [24]. Regarding selenium, selenium yeast supplementation for cows in the late gestation period can improve the antioxidant status and the immune response after calving [25]. Additionally, supplementation with hydroxy-selenomethionine, compared to selenium yeast at the same supplementation level, improved milk quality by reducing the somatic cell count [26]. The possibility of improving the antioxidant response with selenium yeast and selenomethionine supplementation is also indicated [17].

Another benefit that zinc and selenium supplementation can generate is the nutritional fortification of milk, with increased mineral levels. This effect is well reported for selenium supplementation at increasing levels and using organic sources [17,27]. There are indications that zinc supplementation, under the same conditions mentioned for selenium, increases the concentration of zinc in milk [11,28] and even in colostrum [23]. This process makes it possible to promote a differential attribute for marketed milk and reinforce the nutraceutical appeal since zinc and selenium are involved with the immune system and are essential for maintaining a good immune response [29,30]. However, it is worth noting that it is a challenge to adjust supplementation protocols and the form of the micromineral used to meet the nutritional requirements of animals and benefit consumers of dairy products [27].

Therefore, we hypothesized that supplementation of zinc and selenium in organic forms is capable of improving the health status of cows in the final third of lactation by stimulating immunity and antioxidant response, as well as improving milk quality, also resulting in an increase in zinc and selenium levels in milk. Thus, the objective of the present study was to evaluate whether supplementation of organic zinc and selenium in late lactation dairy cows has a positive effect on immunity, oxidative status, milk quality (especially mineral levels), biochemical and hematological parameters, and lactation persistence.

## 2. Materials and Methods

This study was conducted at the Experimental Farm of the Higher Education Center of the West of the University of the State of Santa Catarina in Guatambu, Santa Catarina, Brazil. The Ethics Committee approved the experimental protocol (2049250923) on the Use of Animals of the University of the State of Santa Catarina.

### 2.1. Minerals

The organic minerals used were ZINC 22^®^ and SELENIUM 5000^®^, two commercial products from Olmix Group (Brehan, France). ZINC 22^®^ presented zinc in the form of chelated zinc amino acid, with a nonspecific amino acid and a zinc content of 22%. SELENIUM 5000^®^ presented selenium as a selenium amino acid complex with a nonspecific amino acid and a selenium content of 0.5%. In the amino acid profile, the four main ones were glutamic acid, aspartic acid, leucine, and arginine. Both presented methionine as one of the last three amino acids in the profile, ordered by the percentage of presence, among up to 19 amino acids.

### 2.2. Animals and Installation

Twenty primiparous Jersey cows in the final third of lactation were used in this study (days in lactation 311 ± 54, average production in the last seven days 14.88 ± 1.82 kg, and days in gestation 196 ± 8.20). At the end of the experiment, the cows were dried off (reduced to one milking/day and feeding for three consecutive days, followed by applying dry cow tubes to each teat; no more milking until partum). The animals, housed in a Compost Barn facility, were milked twice a day using a DeLaval VMS™ V300 robotic milker in a guided system, to which they had access from 5:00 to 8:30 and from 15:00 to 17:00. The facility had manually activated fans to control the thermal environment.

### 2.3. Experimental Design and Diet

The study lasted 28 days, with the first 14 days considered an adaptation period. The design was completely randomized with different numbers of replicates. The cows were divided into three groups according to the average production in the previous seven days, days in lactation (DL) and days in gestation: Control (n = 6)—no supplementation in organic forms; Zinc (n = 7)—zinc supplementation in the form of zinc amino acid chelate (40.43 mg zinc/kg dry matter; or 488.39 mg/animal/day); and Selenium (n = 7)—selenium supplementation in the form of selenium amino acid complex (0.89 mg selenium/kg dry matter; or 10.75 mg/animal/day).

The basal diet was formulated to meet the nutritional requirements based on NASEM [9], with the characteristics of the animals used for the formulation shown in Table 1. A commercial vitamin-mineral nucleus (Vitamix^®^ Bovimix Lactation M BIO ST) was used with most minerals, including zinc and selenium, in inorganic forms (zinc sulfate and sodium selenite). The aim was to keep the zinc and selenium levels as close as possible to the nutritional requirements; however, the zinc levels slightly exceeded the requirement. Despite this, using a commercial nucleus was maintained due to the complexity and high cost of developing a specific nucleus for the study.

Organic minerals were added to the concentrate for supplementation of the Zinc and Selenium treatments. The basal diet fed to the Control contained 54.82 mg of zinc (Zn)/kg of dry matter (DM) and 0.3 mg of selenium (Se)/kg of DM. The supplementation level used for the Zinc group was based on Chen et al. [23], where the supplementation level of 40 mg of Zn/kg of DM was used as a reference. The supplementation level of the Selenium group was based on Hachemi et al. [26], considering that the selenium requirement was 0.3 mg/kg DM [9]. It is noteworthy that this level did not exceed the maximum tolerated selenium level of 5 mg/kg and zinc levels of 500 mg/kg of DM [31].

The cows were fed three times a day. The first two feedings were carried out in individual feeders at the same time as milking, starting at 7:40 and 15:00, respectively, and lasting approximately two hours each. The third feeding began at 18:00 when approximately 30 kg of hay were distributed in six automatic feeders (Intergado^®^, Ponta, Betim, Minas Gerais, Brazil) and allowed free consumption. Additionally, during milking, the animals had access to up to 1.2 kg/day of pelleted concentrate specifically for use in robotic milking, Nutrialfa^®^ Bovino Robô AP (CooperAlfa, Bom Jesus, Santa Catarina, Brazil). The centesimal composition of the diets provided in the individual feeders and the levels of minerals of interest in the total diets are shown in Table 2.

### 2.4. Data and Sample Collection

Daily milk production, pelleted concentrate intake, and body condition score (BCS) of each animal were automatically measured and recorded by the robotic milking system. Daily BCS was determined by capturing three-dimensional images of the cow’s loin and rump region with a camera (DeLaval BCS™, Delaval do Brasil, São Paulo, Brazil) positioned at the robot’s exit. Using the images, the robot system classified BCS on a scale of 1 to 5, with 1 representing leaner animals and 5 representing fatter animals. In addition, feed leftovers from individual feeders were weighed and recorded after the second feeding of the day (animals always occupied the same feeder). Hay supply and consumption from automatic feeders (Intergado^®^) were automatically recorded.

Blood, milk, and feed samples were collected on days 1, 14, and 28. Blood was collected from the coccygeal vein, using a holder and needles for collection through vacuum tubes (Vacuplast^®^, Cral, Cotia, São Paulo, Brazil), with 4 mL tubes containing anticoagulant (EDTA K3) for hemogram and 10 mL tubes containing clot activator for serum separation. A blood collection was also performed prior to the start of the experiment to separate serum and measure zinc and selenium levels. Blood samples were stored below 5 °C until arrival at the laboratory. The hemogram was performed immediately after the material arrived at the laboratory. To separate serum, the tubes were centrifuged in a tube centrifuge (QUIMIS^®^, model Q222T, Diadema, São Paulo, Brazil) at 7000 rpm for 10 min at room temperature. The serum was stored in microtubes at −20 °C until analysis.

Milk collections, individualized for each animal, were performed in specific tubes using the milking robot’s collector. A Brononata^®^ (Laborclin, Pinhais, Brazil) preservative tablet was added to the samples intended for milk quality and was stored at 4 °C until sent to the laboratory. Those intended for fatty acid profile and mineral concentration analyses collected only on day 28 were stored in microtubes and kept at −20 °C until analysis.

Regarding the feed, samples were collected from each of the concentrates, silage, hay, pelleted concentrate, and the total basal diet. The three samples of each feed were stored at −20 °C, with subsequent thawing and homogenization to obtain a representative sample of the period from the three collected. Pre-drying was carried out in an oven with forced air circulation at 55 °C for 72 h and subsequent grinding in a Wiley mill (Marconi^®^, model MA340, Marconi, Piracicaba, Brazil), using a 1 mm mesh sieve.

### 2.5. Analysis of Feed Composition

The weight of the samples was measured before and after pre-drying to determine the partial DM content. The ground samples were kept in a forced ventilation oven at 105 °C to finalize the DM determination until completely dry. The mineral matter was quantified by storing the samples in a muffle furnace at 600 °C until the total combustion of the organic matter. The crude protein content was determined by micro-Kjeldahl based on the official method—AOAC 2001.11 [32]. The quantification of ether extract, performed in an automatic fat extractor (VELP Scientifica^®^, model SER 158, Usmate, Italy), followed the official method—AOAC 2003.05 [32], replacing diethyl ether with petroleum ether. Neutral detergent fiber (NDF) and acid detergent fiber (ADF) determinations were performed in a Fiber Analyzer (ANKOM^®^ 200, Ankom, NY, USA) following the equipment methodologies (except for the non-use of α-amylase and the replacement of ANKOM^®^ filter bags by bags made of non-woven fabric), which were based, respectively, on the method described by Van Soest et al. [33] and the official method—AOAC 973.18 [32]. The composition of the basal diet is displayed in Table 3. Furthermore, the composition of silage, hay, and pelleted concentrate supplied in the milking robot is displayed in Table S1 (Supplementary Information).

### 2.6. Zinc and Selenium Levels in Blood Serum, Milk, and Concentrates

Sample preparation was performed by microwave-assisted wet decomposition (MAWD) using an Ethos Easy™ decomposition system (Milestone™, Milan, Italy) equipped with a rotor with 44 PTFE-TFM vessels with an internal capacity of 100 mL (MAXI-44 rotor, Milestone™). The decomposition and analysis of the concentrate samples were performed in triplicate, while the serum and milk samples were analyzed in duplicate due to the low sample volume. Approximately 250 mg of the sample were weighed directly into the decomposition vessels. Then, 6 mL of 14.4 mol/L HNO_3_ distilled below the boiling point was added. The vessels were closed and inserted into the rotor. An irradiation program recommended by the manufacturer for biological samples was used, consisting of three steps: (i) 20 min ramp to 200 °C (1800 W); (ii) 20 min stay at 200 °C (1800 W); (iii) cooling to 50 °C (without microwave irradiation). After the MAWD procedure, the digests were collected and made up to 20 mL with ultrapure water.

The selenium and zinc determination was performed by inductively coupled plasma mass spectrometry (ICP-MS) using an Elan DRC II^®^ spectrometer (Perkin Elmer^®^, Ontario, Canada) equipped with a concentric pneumatic nebulizer, cyclonic nebulization chamber and torch with a 2 mm internal diameter quartz injector tube. High-purity argon (99.998%, White Martins^®^, Chapecó, Brazil) was used for plasma generation, as well as for nebulization and auxiliary gases. The operating conditions of the ICP-MS equipment were: (a) RF generator power: 1300 W; (b) air flow rates: main 15 L/min, auxiliary 1.2 L/min and nebulization 1.02 L/min; (c) platinum sampling and skimmer cones were used; (d) monitored isotopes (*m*/*z*): ^82^Se, ^64^Zn, and ^66^Zn. The methodology for selenium and zinc determination was based on Pereira et al. [34] and Cruz et al. [35]. The results for serum and milk were presented in µg/g, while those for concentrates were adjusted to be expressed in mg/kg of DM of the concentrate and mg/kg of DM of the total diet. The mineral levels in the diets are shown in Table S2.

### 2.7. Hemogram

The blood count was performed on an automatic hematology analyzer (EQUIP VET^®^ 3000, Equip, Itatiba, São Paulo, Brazil), focusing on the erythrocyte count (×10^6^/µL), total leukocyte count (differentiated lymphocytes, granulocytes, and monocytes; ×10^3^/µL) and platelets (×10^3^/µL), as well as the hemoglobin concentration (mg/dL) and hematocrit percentage (%).

### 2.8. Serum Biochemistry

Serum biochemistry was performed using an automatic analyzer (Zybio^®^ EXC 200, Equip, Itatiba, São Paulo, Brazil) and commercial kits (Analisa^®^, Belo Horizonte, Brazil). The variables analyzed were the activity of the enzymes creatine kinase (CK) and cholinesterase (U/L), as well as the levels of iron (µg/dL), total protein, albumin (g/dL), cholesterol, calcium, phosphorus, magnesium and C-reactive protein (mg/dL), fructosamine (µmol/L), urea (g/dL) and ferritin (µg/L). Globulin levels were obtained mathematically (total protein—albumin).

For the proteinogram, sodium dodecyl sulfate-polyacrylamide gel electrophoresis (SDS-PAGE) was performed according to the technique described by Fagliari et al. [36] using mini-gels (10 × 10 cm). The gels were stained with Coomassie blue and photographed to identify and quantify the protein fractions using LabImage 1D software (Loccus Biotechnology, Cotia, São Paulo, Brazil). A standard containing fractions with molecular weight between 10 and 250 KD (Kaleidoscope—Bio-Rad) was used as a reference. The total protein content previously obtained using the biuret technique was used as a reference for quantification. Immunoglobulin A (IgA), heavy chain immunoglobulin (Ig-heavy chain), ceruloplasmin and haptoglobin concentrations were analyzed and expressed as g/dL.

Adenylate kinase (AK) activity was measured with a coupled enzymatic assay using hexokinase (HK) and glucose 6-phosphate dehydrogenase (G6PD), according to Dzeja et al. [37] with adaptations described by Fortuoso et al. [38]. The reaction mixture contained 100 mM of KCl, 20 mM of HEPES, 20 mM of glucose, 4 mM of MgCl_2_, 2 mM of NADP+, 1 mM of EDTA, 4.5 U/mL of HK, 2 U/mL of G6PD, and 20 μL of sample. The addition of 2 mM ADP initiated the reaction, and the reduction of NADP+ was evaluated at 340 nm for 3 min in a spectrophotometer. The results were expressed as nmol of ATP formed per min per mg of protein.

Pyruvate kinase (PK) activity was assayed as described by Leong et al. [39] with adaptations described by Fortuoso et al. [38]. The incubation medium consisted of 0.1 M of Tris/HCl buffer, pH 7.5, 10 mM of MgCl_2_, 0.16 mM of NADH, 75 mM of KCl, 5.0 mM of ADP, 7 U of L-lactate dehydrogenase, 0.1% (*v*/*v*) of Triton X-100, and 20 μL of the sample in a final volume of 500 μL. After 10 min pre-incubation at 37 °C, the reaction was started by adding one mM phosphoenolpyruvate (PEP). Results were expressed as nmol of pyruvate formed per min per mg of protein.

### 2.9. Oxidative Status

The oxidative status variables evaluated in blood serum were reactive oxygen species (ROS), thiobarbituric acid-reactive substances (TBARS), myeloperoxidase activity (MPO), total thiols (PSH), non-protein thiols (NPSH) and ascorbic acid or vitamin C. The analyses were performed in triplicate, using specific biochemical methodologies. Both colorimetric and fluorimetric readings were made in Varioskan™ LUX (Thermo Scientific™, Waltham, MA, USA).

The formation of ROS was estimated by the fluorimetric protocol established by Ali et al. [40]. A total of 10 µL of serum was incubated with 10 µL of 2′,7′-dichlorofluorescein diacetate (DCFH-DA, 7 μM) and 240 µL of PBS. After 30 min of incubation at 37 °C, the final product of DCFH-DA oxidation, the dichlorofluorescein (DCF), was measured. The fluorescence emission intensity was read with an emission of 525 nm and an excitation of 488 nm. The results are expressed as a percentage (%) of fluorescence intensity relative to control.

Lipoperoxidation is a highly rapid reaction formed by the breakdown of polyunsaturated fatty acids, which are usually measured by their products, mainly TBARS, among which malondialdehyde (MDA) is the primary one [41]. To evaluate this product, the reaction of thiobarbituric acid (TBA) with serum samples was used, which, in the presence of MDA, results in a pink product that can be read at 532 nm. Briefly, 20 µL of samples were mixed with 55 µL of distilled water, 100 µL of orthophosphoric acid (0.2 M), and 25 µL of TBA (0.1 M). A spectrophotometric reading was taken after 45 min of incubation at 37 °C. Results were expressed in nM MDA/mL.

MPO is a heme enzyme produced by inflammatory mediators and released from leukocytes at the injury site; therefore, MPO reflects the activation of neutrophils and lymphocytes. MPO catalyzes the reaction of chloride ions with H_2_O_2_ to generate large amounts of hypochlorous acid (HOCl), a ROS that further reacts to generate singlet oxygen and hydroxyl radicals. In the presence of H_2_O_2_ as an oxidizing agent, MPO catalyzes the oxidative coupling of phenol and 4-aminoantipyrine (AAP), originating a colored product, quinoneimine, with a maximum absorbance of 492 nm [42]. The MPO activity was analyzed using a modified peroxidase system, with a mixing of 12 µL of serum sample with 148 µL of AAP in phenol solution (AAP 2.5 mM; phenol 20 mM) and 17 µL of H_2_O_2_ solution (17 mM). After 30 min of incubation at 37 °C, the system was read spectrophotometrically. The results were expressed as μM of quinoneimine per mg of protein produced in 30 min (μMq/mg/30 min).

Both thiol contents were determined according to Ellman [43] with adjustments. For PSH assay, 30 µL of supernatant in a 96-well plate was added to 200 µL of potassium phosphate buffer (PPB) (1 M, pH 6.8) and 20 µL of 5,5ʹ-dithiobis (2-nitrobenzoic acid) (DTNB) with immediate reading. For NPSH, the samples were previously deproteinized to 10% with an equal sample volume of trichloroacetic acid (TCA), and the remaining supernatant was used. Then, 40 µL of supernatant was mixed with 260 µL of PPB and 15 µL of DTNB with immediate reading. All readings were measured at 412 nm. The results were expressed in µM/L having a cysteine standard curve as the parameter.

The ascorbic acid content was measured according to Jacques-Silva et al. [44], with an absorbance reading at 520 nm. Briefly, 200 μL of serum samples were first deproteinized with an equal volume of trichloroacetic acid (TCA, 10%). After, 100 μL of remaining supernatant was mixed with 25 μL of distilled water, 25 μL of TCA (13.3%), and 20 μL of 2,4-dinitrophenylhydrazine (DNPH), followed by 2 h of incubation at 37 °C. At the endpoint, an orange-red compound product was generated, proportional to ascorbic acid content, which was expressed as µg/dL according to ascorbic acid standard.

### 2.10. Milk Quality

Milk quality analyses were performed by the Centralized Milk Analysis Laboratory of the Paraná Dairy Herd Analysis Program, accredited by the Ministry of Agriculture, Livestock and Food Supply. Fourier transform mid-infrared spectrometry determined the total protein, lactose, fat, total solids, and milk urea nitrogen contents, expressed as percentages, according to ISO 9622/IDF 141:2013 [45]. The somatic cell count (SCC), expressed as ×103/mL, was determined by flow cytometry according to ISO 13366-2/IDF 148-2:2006 [46]. Additionally, it should be noted that one of the animals in the Zinc treatment was removed from the SCC evaluation since it had a history of high SCC compared to the other animals in the herd.

### 2.11. Fatty Acid Profile in Milk and Diet

Lipid extraction was based on the Bligh and Dyer [47] method based on organic solvent extraction using methanol and chloroform. Briefly, per 3.0 mL of sample, 10.0 mL of methanol, 5.0 mL chloroform, and 1.3 mL water were added and shaken for 60 min. A total of 5 mL chloroform and 5 mL sodium sulfate solution (1.5%) were added and shaken for 30 min. The mixture had a final ratio 2:2:1.8 (methanol:chloroform:water). The samples were centrifuged at 168× *g* for 10 min for phase separation.

Lipids were subjected to transesterification to obtain the fatty acid methyl esters (FAME), according to Hartman and Lago [48]. The extracted lipids were added to 1 mL of 0.4 M KOH methanolic solution in a test tube and shaken in a vortex for 1 min. Samples were kept in a water bath for 10 min at boiling point. Subsequently, they were cooled at room temperature, and 3 mL of 1 M H_2_SO_4_ methanolic solution was added, shaken in a vortex, and maintained in a water bath for 10 min. After cooling, 2 mL of hexane was added and centrifuged at 2000 rpm for 10 min.

FAME dissolved in hexane were analyzed in a gas chromatograph equipped with a flame ionization detection (FID) Thermo Scientific™, model TRACE™ 1310 (Austin, TX, USA) and separated in an RT™ 2560 (100 m × 0.25 mm × 0.20 μm of thickness film, Restek™, Bellefonte, PA, USA) chromatography column. The carrier gas used was hydrogen at a constant flow of 1.5 mL/min. The injector remained in 20:1 split mode and a temperature of 250 °C. The initial temperature of the column was 50 °C, where it remained for 1 min, increasing to 185 °C at a rate of 15 °C/min, after 195 °C with an increase rate of 0.5 °C/min and then with a rate from 15 °C/min until reaching 250 °C keeping in isotherm for 6 min. The detector remained at a temperature of 250 °C. The FAME compounds were identified by comparison of experimental retention time with those from authentic standards (FAME Mix-37, Sigma Aldrich™, St. Louis, MO, USA). The results were presented as a percentage of each fatty acid (FA) identified in the lipid fraction, considering the chain size equivalent factor of FAME for FID and conversion factor of ester to the respective acid, according to Visentainer [49]. The fatty acid profile of the basal diet is presented in Table S3 (Supplementary Information).

### 2.12. Productive Performance

The DMI was calculated by adding the consumption of the partial diet in the individual feeders, the hay in the automatic feeders, and the pelleted concentrate in the milking robot. These were converted to DM based on the results obtained in the feed analyses. The milk production corrected for 4% fat (FCM) was calculated as follows: FCM = 0.4 × Milk production + 15 × Fat production [50], in which the variables were expressed in kilograms. Feed efficiency was obtained by the calculation: Feed efficiency = (Milk production)/DMI. Furthermore, lactation persistence was obtained by the equation Lactation persistence (%) = {1 − [(MPi − MPf) × (30/(f − i))/MPi]} × 100, in which MPi is the initial milk production, MPf is the final milk production, i is the initial day (day zero), and f is the final day (28). Additionally, it should be noted that two animals, one from the Zinc group and one from the Selenium group, were removed from the milk production analysis to homogenize the initial average milk production of the groups.

### 2.13. Statistical Analysis

All data were analyzed using the ‘MIXED procedure’ of SAS^®^ (SAS Inst. Inc., Cary, NC, USA; version 9.4), with Satterthwaite approximation to determine the denominator degrees of freedom for the test of fixed effects. The milk production, milk fatty acid, lactation persistence, and feed efficiency were tested for fixed effects of treatment using animals (treatment) as random effects. The data of milk production, all blood, and milk results were analyzed as repeated measures and were tested for fixed effects of treatment, day, and treatment × day, using animal (treatment) as random effects. The d1 results were included as an independent covariate. The first-order autoregressive covariance structure was selected according to the lowest Akaike information criterion. Means were separated using the PDIFF method (Tukey test), and all results were reported as LSMEANS followed by SEM. Significance was defined when *p* ≤ 0.05, and tendency when *p* > 0.05 and ≤0.10.

## 3. Results

### 3.1. Zinc and Selenium in Blood Serum and Milk

The results of the serum and milk mineral levels are presented in Table 4. The results observed for Se and Zn in milk and serum, respectively, were below the limits of quantification of the analytical method. The selenium concentration in the blood serum on day 28 was higher in the Selenium group than in the Control group; nevertheless, compared to the Control group, it was lower in the Zinc group (*p* = 0.01). There was an effect for the level of zinc in the milk to be higher in the group supplemented with zinc in the organic form (*p* = 0.05).

### 3.2. Hemogram and Serum Biochemistry

In the hemogram, with results in Table 5, there was a treatment x day interaction in the lymphocyte count (*p* = 0.01). On day 14, the treatment supplemented with selenium was higher than the Control. On day 28, it was higher in both treatments that received organic mineral supplementation than the Control. The effects of the treatments on the leukocyte count (*p* = 0.09) showed a tendency to be higher in the groups supplemented with zinc and selenium than in the Control group. The erythrocyte, platelet, granulocyte, monocyte counts, hemoglobin concentration, and hematocrit percentage did not differ between treatments.

The serum biochemistry results are also presented in Table 5. A treatment x day interaction was detected in serum iron and globulin levels (*p* = 0.01) and cholinesterase activity (*p* = 0.03). The treatments also affected serum fructosamine levels (*p* = 0.05). On day 14, serum iron levels were lower in the group supplemented with organic zinc than the others. On day 28, they were lower in both groups supplemented with organic minerals than the Control. Cholinesterase activity was lower in the Zinc and Selenium treatments than in the Control on day 14. It remained lower on day 28 only in cows in the treatment supplemented with organic selenium compared to the Control. Serum globulin levels differed between treatments only on day 14, in which the Selenium treatment had a higher globulin level than the Control. Furthermore, fructosamine levels were lower for both supplemented groups when compared to Control. The concentrations of albumin, total protein, cholesterol, urea, calcium, phosphorus, magnesium, C-reactive protein, and ferritin did not differ between treatments.

**Figure 1:**
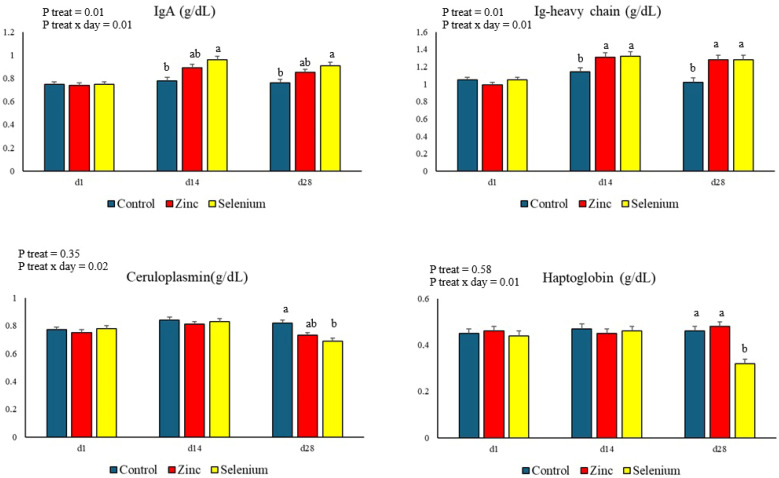
Proteinogram in blood serum of Jersey cows supplemented with zinc and organic selenium. Note 1: Different letters in the same day show statistical differences between groups (*p* ≤ 0.05). Note 2: Ig = Immunoglobulin.

The results of the proteinogram are presented in Figure 1. There was a treatment x day interaction in the IgA and Ig-heavy chain concentrations (*p* = 0.01). On days 14 and 28, the IgA concentration was higher in the Selenium group than in the Control group, as well as the Ig-heavy chain concentration was higher in both treatments that received organic mineral supplementation than the Control. On day 28, ceruloplasmin (*p* = 0.02) and haptoglobin (*p* = 0.01) concentrations were lower in the Selenium group than in the Control group.

In the biochemistry of energy metabolism, with results presented in Table 6, there was a treatment × day interaction in the activities of the enzymes CK, AK, and PK (*p* = 0.01). On day 14, CK activity was lower in the Zinc group than in the Control, while on day 28, it was lower in both groups supplemented with organic minerals. AK and PK activities were higher in both supplemented groups than in the Control on day 14. On day 28, AK activity remained higher than the Control group in both supplemented groups, but PK activity remained higher than the Control group only in the Zinc group.

### 3.3. Oxidative Status

The results of the oxidative status are presented in Table 7. A treatment x day interaction was observed in ROS, MPO activity (*p* = 0.01), and TBARS levels (*p* = 0.05). On days 14 and 28, the supplemented treatments presented a lower ROS level than the Control. Furthermore, MPO activity and TBARS levels on day 28 were lower in the Zinc and Selenium treatments than in the Control. The serum levels of PSH, NPSH, and ascorbic acid/vitamin C were not influenced by the supplementation of these organic minerals at the levels used.

### 3.4. Milk Quality

Regarding milk quality (Table 8), the treatments did not influence total protein and lactose percentages. The MUN was affected by the treatments (*p* = 0.05), and the group of cows supplemented with organic selenium presented a lower MUN than the Control. There was a treatment × day interaction in the percentage of fat (*p* = 0.05) and in the SCC (*p* = 0.01), as well as a trend in the percentage of total solids in the milk (*p* = 0.08). On days 14 and 28, the fat percentage was lower in the treatment supplemented with selenium in the organic form than in the Control. Additionally, there was a trend for both supplemented groups to present a lower percentage of total solids than the Control. Furthermore, on day 14, the SCC was lower in the Zinc treatment than in the Control. On day 28, the Zinc and Selenium groups presented a lower SCC in the milk than in the Control.

### 3.5. Fatty Acid Profile in Milk

The results of the milk fatty acid profile are shown in Table 9. There was an effect of treatment on caproic acid (C6:0), lauric acid (C12:0) (*p* = 0.05), myristic acid (C14:0), and the sum of saturated fatty acids (∑ SFA) (*p* = 0.01), which were lower in the group supplemented with selenium when compared to the Control. The treatment also influenced the percentage of palmitic acid (C16:0), which tended to be lower in both supplemented groups (*p* = 0.08). Additionally, elaidic acid (C18:1 n9t) (*p* = 0.02), the sum of monounsaturated fatty acids (∑ MUFA) (*p* = 0.032) and polyunsaturated fatty acids (∑ PUFA) (*p* = 0.06, trend), as well as the sum of omega 6 (∑ ω6) (*p* = 0.045), were affected by the treatments. In these parameters, the percentage of fatty acids or sum was higher in the group supplemented with organic selenium than in the Control. In addition, oleic acid (C18:1 n9c) (*p* = 0.01) and the sum of unsaturated fatty acids (∑ UFA) (*p* = 0.001) were influenced by mineral supplementation, in which the percentage was higher in the treatment that received organic selenium compared to the Control. Furthermore, the ratio between unsaturated fatty acids/saturated fatty acids (UFA/SFA) was higher in both supplemented groups than in the Control (*p* = 0.01). The treatments did not influence the other fatty acids, the sum of omega 3 fatty acids (∑ ω3), and the omega 6/omega 3 ratio (ω6/ω3).

### 3.6. Productive Performance

The results of productive performance are shown in Table 10. Organic mineral supplementation did not affect milk production, FCM, lactation persistence, and DMI. On the other hand, the BCS of the treatment supplemented with zinc was higher than the Control between days 15 and 28 (*p* = 0.05). In addition, feed efficiency from days 15 to 28 tended to be higher in treatments supplemented with organic minerals compared to the Control (*p* = 0.07). As DMI differed from that estimated in the formulation (12.08 kg/animal/day), the expected and effectively supplemented mineral intakes of zinc and selenium were shown in Table 10 in mg/animal/day, as well as the zinc requirements were recalculated, since they are affected by DMI and milk production [9], which also differed from that expected in the formulation (15 kg/animal/day). The values used for recalculation were those shown in Table S4, and the calculation was made for each treatment based on the total period (days 1 to 28). The selenium requirement was not recalculated since it was set at 0.3 mg/kg DM [9].

## 4. Discussion

Micromineral supplementation strategies with organic minerals vary. The differences range from the level of supplementation to the organic form used, since the mineral can be bound to different molecules. The present study evaluated the effects of supplementation with chelated zinc amino acid and selenium amino acid complex compared to zinc and selenium supplementation in conventional forms (zinc sulfate and sodium selenite). The main findings were related to the immune system, inflammatory indicators, oxidative status, and milk quality.

The calculated dietary levels of minerals still needed to be achieved. Nevertheless, supplementation in organic forms resulted in supra-nutritional levels for the Zinc and Selenium groups. However, the Control and Zinc groups presented a selenium concentration of 0.28 mg/kg DM in the total diet, slightly lower than the nutritional requirement of 0.3 mg/kg DM [9]. Furthermore, considering the DMI of the Control (11.7 kg) and Selenium (11.2 kg) groups and the zinc concentration in the diets of 43.89 mg/kg DM, the zinc supply was, respectively, 513.51 and 491.57 mg/animal/day. The zinc requirement of 602.00 mg/animal/day (DMI 12.08 kg/day; 49.83 mg of Zn/kg of DM), calculated for the animals used in the study based on NASEM [9] (with the characteristics mentioned in the methodology) did not materialize, since DMI and milk production differed from what was expected. Recalculating the zinc requirement based on the results obtained, the requirement would be 546.50 mg/animal/day for the Control group (DMI 11.7 kg and milk production 12.7 kg) and 538.00 mg/animal/day for the Selenium group (DMI 11.2 kg and milk production 12.9 kg). Therefore, it is inferred that the Control and Selenium groups did not meet their zinc requirements. Goff [19] reported that the normal serum zinc concentration is 0.7 to 1.3 µg/mL, with values below 0.4 µg/mL generally classified as a deficiency. However, as already mentioned, the results observed for zinc in blood serum were below the limits of quantification of the analytical method. Therefore, comparing serum zinc levels between the different groups and the reference ranges was impossible.

Selenium concentration in blood serum was higher in the group supplemented with organic selenium. This result agrees with those found by Respati et al. [17], who, in a meta-analysis of 29 studies, indicated that organic sources presented a more significant transfer of selenium from the diet to the blood than inorganic sources. This finding is due to the different absorption or metabolism routes of selenium from organic and inorganic sources [17,18]. Vendeland et al. [51], when studying the intestinal absorption of different forms of selenium in rats, stated that an organic form, selenomethionine, had a faster and more significant absorption than inorganic sources. In addition, the total absorption of selenate was 40% greater than that of selenite [51]. In our study, the inorganic form used was sodium selenite. Weiss [52] indicated that selenite appears to be absorbed mainly by passive diffusion, selenate by an active transport system, and selenomethionine by the methionine transport system. Wichtel et al. [53] stated that serum selenium concentrations ≥ 0.07 µg/mL are recommended, and ≤ 0.03 µg/mL are classified as a deficiency. Villar et al. [54] found a serum selenium value in Holstein cows of 68.4 ± 17 ppb, or 0.0684 ± 0.017 µg/g. The values found in the present study for the Control (0.066 µg/g) and Selenium (0.087 µg/g) groups on day 28 meet or exceed the range of Villar et al. [54]. Furthermore, interestingly, serum selenium concentration was lower in the group supplemented with organic zinc compared to the Control. The serum selenium level of the Zinc group was < 0.030 µg/g, below the average range found by Villar et al. [54]. However, to the best of our knowledge, there are no reports of an antagonistic relationship between these minerals. Therefore, this result requires future studies.

The zinc level in milk was higher in the group supplemented with organic zinc. The results of Cai et al. [28], who supplemented Holstein cows with an initial DL of 158 with zinc methionine, in addition to the basal diet already meeting the zinc requirement, indicated that the zinc level in milk was higher in the group supplemented with the organic form, which reinforces the result found in our study. Oconitrillo et al. [11], who provided two dietary levels of zinc in the form of zinc methionine to Holstein cows in the first third of lactation, indicated that the zinc concentration in milk tended to be higher in the group with the highest level of supplementation. Sauer et al. [55], when studying an in vitro model, indicated that zinc binding to amino acids causes it to be absorbed by amino acid transporters. This less saturable absorption pathway reduces the effect of antagonistic factors. With these studies in mind, the understanding of the higher zinc level in milk is expanded.

A possible positive influence of organic zinc supplementation on the immune system was the increase in lymphocyte counts and, consequently, a tendency for an increase in leukocyte counts. This hypothesis is reinforced by the increase in globulins, with emphasis on immunoglobulins that are produced by B lymphocytes, responsible for stimulating the humoral response, which is desired in production animals, as in this type of immune response the energy expenditure is small. The involvement of zinc [9,22,56] and selenium [25,26,57] in the immune system is already documented. In contrast, there are reports of different organic zinc supplementation strategies not altering lymphocyte and leukocyte counts [11,15,16,28]. On the other hand, the participation of zinc in the control of homeostasis and lymphocyte function is known [29]. Homeostasis is regulated by maintaining the proliferation of these cells [58] and preventing apoptosis [59], including cell activation [58]. Given this, it is possible to understand the result found. Likewise, there are reports of no change in lymphocyte count with different strategies of selenium supplementation in organic form [26,60]. However, Li et al. [60] reports the influence of leukocyte count and Hachemi et al. [26] on neutrophil, monocyte, basophil, and leukocyte counts. It is known that some selenoproteins have immunological functions [26,57] and that selenium affects the activation and proliferation of lymphocytes [61]. Thus, the mechanisms and conditions that determine the influence of selenium on different types of leukocytes must be elucidated.

Zinc and selenium supplementation resulted in a higher immunoglobulin concentration. The Ig-heavy chain concentration was higher in both groups supplemented with organic minerals, reinforcing the interpretation that organic zinc and selenium supplementation positively boosted the immune system. In a more specific way, the organic selenium supplementation increased the IgA concentration. IgA plays a fundamental role in the immune system of neutralizing pathogens, mainly on mucosal surfaces [62]. Gong et al. [7] also reported an increase in IgA concentration in dairy cows supplemented with organic Se, with the result found in only one of the blood samplings (day 30) and comparing supplementation at the same level of Se yeast and inorganic Se (0.3 mg Se/kg DM). To the best of our knowledge, the mechanism that determines the influence of selenium on IgA production still needs to be understood. Apparently, organic zinc supplementation has no effect on IgA concentration, which is confirmed by Chen et al. [23] and Oconitrillo et al. [11].

The use of organic selenium at a supra-nutritional level determined the increase in the serum concentration of globulins. Globulins are a class of plasma proteins that can be divided into three fractions (α, β, and γ) with varied functions [63]. The serum concentration of globulins can be interpreted as an indication of the response or status of humoral immunity [64]. However, as it includes the main positive acute phase proteins [63], it can also be interpreted as an inflammatory indicator [65]. Considering the other responses to selenium supplementation, which were positive for the immune system, oxidative status, and anti-inflammatory indicators, the present result can be interpreted as an indication of the stimulation of immunity. However, there are reports that selenium supplementation does not influence the globulin concentration of lactating cows [60,66]. Nevertheless, these studies used supplementation strategies that differed from those used in the present study, mainly due to lower levels of selenium in the diet. In order to confirm the result found, Mehdi and Dufrasne [13] indicated that globulins α and β are linked to selenium.

Confirming the interpretation of the increase in serum globulin concentration, the organic selenium supplementation reduced the acute phase proteins, ceruloplasmin and haptoglobin, concentrations. As these proteins are inflammatory markers, it can be said that there was a beneficial influence in terms of inflammation. It is known that the serum concentration of these proteins increases linearly with increasing SCC [67], and that mastitis, whether clinical or subclinical, causes an increase in the serum concentration of these proteins [68]. Supplementation in organic forms also decreased SCC as shown further on. Although organic zinc supplementation was not able to reduce the concentrations of ceruloplasmin and haptoglobin, the SCC, on day 28, of the organic zinc supplemented group was even lower than that of the selenium supplemented group, which indicates the health of the mammary gland and the absence of subclinical inflammation.

Supplementation of both minerals induced a reduction in cholinesterase activity, which is a marker of low-grade inflammation [69]. Cholinesterase can be subdivided into acetylcholinesterase and butyrylcholinesterase, and it is already known that when the activity of these enzymes increases, there is a consequent reduction in the levels of acetylcholine, which is an anti-inflammatory molecule [69]. Therefore, the reduction in cholinesterase activity can be interpreted as beneficial due to the increase in anti-inflammatory activity mediated by acetylcholine. Proof of this is that researchers found that cows infected with *Neospora caninum* had higher serum cholinesterase activity than seronegative cows [70]. In an additive manner, it is known that zinc substances can inhibit cholinesterase activity [71] as well as selenium substances [72]. However, a study that evaluated the supplementation or not of selenium in conventional inorganic forms or hydroxy-selenomethionine at up to 0.5 mg Se/kg DM in lactating cows indicated no change in cholinesterase activity [73]. However, the level of selenium used was lower than that used in our study, and the organic form used differed. Studies that evaluated the activity of this enzyme in cows supplemented with different forms of zinc have yet to be discovered.

Zinc and selenium supplementation also resulted in reduced CK activity. CK is an enzyme related to the synthesis and utilization of adenosine triphosphate (ATP) [74] and, therefore, to the maintenance of energy homeostasis [75]. Extracellular ATP is known to be a pro-inflammatory mediator [76,77]. Thus, lower CK activity translates into lower ATP availability in the extracellular environment and, consequently, a reduced pro-inflammatory response. Reports of zinc supplementation’s influence on lactating cows’ CK activity are unknown. On the other hand, studies on the influence of selenium supplementation reported no change in CK activity [66,73,78]. Tong et al. [79] reports that zinc has different effects, dependent on the mineral level, on CK activity. Enzyme inactivation and denaturation were reported in incubation with levels higher than 0.1 mM zinc. Thus, it is understood why supra-nutritional zinc supplementation can directly affect reducing CK activity. Furthermore, selenium is also related to CK activity; it is known to be elevated in lambs with selenium deficiency myopathy and negatively correlates with glutathione peroxidase (GSH-Px) activity in erythrocytes [80]. GSH-Px is a selenoprotein [9,12]. Given the above, it is understood that supra-nutritional levels of selenium can reduce CK activity.

Although reduced CK activity is beneficial from an inflammatory point of view, it can lead to impaired energy homeostasis due to reduced availability of extracellular ATP. Therefore, it is believed that the increased activity of AK and PK enzymes in both groups supplemented with organic minerals occurred as a regulatory mechanism of energy metabolism to compensate for the lower availability of ATP resulting from lower CK activity. AK is known to play a role in metabolic monitoring, mainly related to cellular energy status, and in signaling, which ensures the efficiency of cellular energy savings [81]. Fortuoso et al. [38], when studying broiler chickens experimentally infected with *Eimeria* spp., discussed the relationship between CK and AK, indicating that increased AK activity can be considered a response to decreased CK activity. This relationship is reinforced by the fact that AK is active under conditions in which CK activity is suppressed [82]. Furthermore, PK catalyzes the conversion reaction of PEP to pyruvate, which generates ATP [83]. Therefore, the proposed interpretation is reinforced.

Organic zinc and selenium improve antioxidant activity, which is identified by reducing ROS, TBARS, and MPO activity. The reduction of ROS can be beneficial, as high concentrations cause irreversible functional changes or even destruction of proteins, lipids, carbohydrates, and nucleic acids [84]. MDA is the main TBARS [41], and the decrease in concentration indicates better antioxidant activity [17]. MPO is an enzyme produced by inflammatory mediators, which catalyzes a reaction that forms ROS [85]. It is known that adequate levels of selenium are involved in maintaining efficient levels of endogenous antioxidants [86]. In an additive manner, the involvement of zinc with the oxidative status is also known, generally associated with its participation in the enzyme Cu-Zn superoxide dismutase (SOD) [9,87]. SOD is a powerful antioxidant enzyme that plays a role in cellular protection against oxidative stress [88]. Confirming the results found, Chen et al. [23] observed a linear increase in the total antioxidant capacity in the blood of Holstein cows with increasing levels of zinc supplementation in the form of zinc methionine. Respati et al. [17], in a meta-analysis, concluded that supplementation in cows with organic forms of selenium increased SOD activity and decreased MDA levels. Thus, the benefit of zinc and selenium supplementation on the oxidative state is evident.

Supplementation in organic forms was also beneficial for mammary gland health, identified by the reduction in SCC. This result agrees with those of stimulation of immunity, anti-inflammatory and antioxidant responses already discussed and known [17,23,26,60,89,90,91]. Kellogg et al. [24] reported, in a review of 12 studies, a reduction in SCC with supplementation, in addition to an inorganic source, of zinc methionine (180 to 400 mg of Zn/cow/day) and the magnitude of the reduction was more significant with supplementation of higher levels of zinc. Hachemi et al. [26] observed a reduction in SCC with organic selenium supplementation in the form of hydroxy-selenomethionine with the highest supplementation level used (0.3 mg Se/kg MN, totaling 1.177 mg Se/kg DM in the total diet). As seen in our study, these results reinforce the hypothesis that zinc and selenium supplementation can benefit mammary gland health.

Organic selenium supplementation also decreased the MUN content. Different organic supplementation strategies for this mineral do not appear to affect this parameter [26,60,66,92]. However, the level and source of supplementation in the present study differ from those mentioned. It is known that selenium is a component of specific proteins, selenoproteins [9,12,14]. However, the mechanism that determines better protein utilization, indicated by the reduction in MUN, is unknown. Furthermore, in the present study, selenium supplementation did not affect urea concentration in blood serum but showed a trend toward improved feed efficiency, discussed later. In this sense, Wang et al. [93] indicated that supplementation of selenium yeast can increase the apparent digestibility in the total tract of crude protein. It is emphasized that the mechanism that determines the influence of selenium on MUN needs to be elucidated.

The reduction in the percentage of fat in milk observed in the Selenium treatment compared to the Control contradicts the results of Respati et al. [17], who reported that selenium supplementation did not affect the fat percentage in milk. In contrast, Sun et al. [78] observed a reduction in the % of fat in milk in early lactation Holstein cows supplemented with hydroxy-selenomethionine (0.3 mg Se/kg DM) compared to the control, which received supplementation of the mineral, at the same level, in the form of sodium selenite. Hu et al. [94] identified, from the liver of mice supplemented or not with selenium, that selenium affects the expression of genes related to cholesterol homeostasis, pancreatic β cell signaling (including metabolic mediators and glucose transporters), and fatty acid metabolism. This influence, mainly on the metabolism of fatty acids, helps to understand the possible relationship between selenium and milk fat production. Since the reduction in fat percentage in milk is undesirable, it should be studied in greater depth to understand better the mechanism that determines this result, and the factors and processes involved.

Undesirably, organic supplementation of zinc and selenium showed a tendency to reduce the total solids content in milk. For selenium, this decrease can be explained by the decrease in the fat content in milk, as discussed above. However, given the unchanged protein, lactose, and fat contents in milk for the zinc treatment (the main components of the solids content), this result still needs to be explained and requires future studies to be understood. In addition to the change in milk fat percentage, organic selenium supplementation modified the milk fatty acid (FA) profile. Ran et al. [95] and Ianni et al. [92] also reported changes in the milk FA profile with selenium supplementation. Ianni et al. [92] used Holstein cows with an average initial DL of 76 days, divided into two groups over 63 days of study: control, with a conventional feeding strategy, and a group supplemented with 0.45 mg Se/kg MN in the form of selenomethionine. Ran et al. [95] used Holstein cows from 30 days pre-partum to 90 days post-partum divided into three groups: control (no supplementation), sodium selenite supplementation (5 mg Se/cow/day) or selenium yeast supplementation (5 mg Se/cow/day). In the present study, reductions in the percentage of lauric (C12:0), myristic (C14:0), and palmitic acids (C16:0; trend) were observed, which were not reported by Ianni et al. [92], as well as the reduction of C14:0 and C16:0 was not observed by Ran et al. [95]. In addition, there was an increase in oleic acid (C18:1 n9c), which was not observed by Ianni et al. [92]. Furthermore, in the present study, SFA was reduced, as reaffirmed by Ianni et al. [92]. There was also an increase in MUFA and a tendency toward an increase in PUFA, which contradicts the results of Ianni et al. [92] and, for MUFA, those of Ran et al. [95], since Ran et al. [95] only observed an increase in PUFA for the group supplemented with organic selenium. Furthermore, the present study observed a reduction in caproic acid (C6:0) and an increase in elaidic acid (C18:1 n9t), ∑ ω6, UFA, and the UFA/SFA ratio, which were not analyzed in the cited studies. The divergences in the profile changes may be due to the different levels and sources of supplementation.

Regarding the FA profile of milk from the treatment supplemented with organic zinc, the results were compared to those of Ianni et al. [96], one of the few studies on the influence of zinc supplementation on the FA profile of milk. Ianni et al. [96] reported a change in the FA profile of milk from Holstein cows with an average initial DL of 78 days and a 49-day study duration. The control received 37 mg of Zn/kg of DM (814 mg/animal/day), and the treatment had an additional 59.1 mg of Zn/kg of DM in the form of zinc oxide (1300.2 mg/animal/day). The increase in the percentage of oleic (C18:1 n9c) and linoleic (C18:2 n6) acids, observed by Ianni et al. [96], was not observed in the present study. Additionally, the increase in MUFA and PUFA, to the detriment of SFA, observed by Ianni et al. [96], was not observed in the present study in the form of changes in the percentages of UFA, MUFA, PUFA, and SFA. However, an increase in the UFA/SFA ratio was observed. On the other hand, the tendency for a reduction in palmitic acid (C16:0), observed in the present study, was not reported by Ianni et al. [96]. Furthermore, Ianni et al. [96] reported an increase in vaccenic (C18:1 trans-11) and conjugated linoleic (rumenic) acids, which were not analyzed in the present study. Similarly to selenium, the divergences in the profile changes may be due to the different levels and sources of supplementation.

The mechanisms of modification in the fatty acid profile in milk induced by zinc and selenium supplementation still need to be better understood. Ran et al. [95], Ianni et al. [92], and Ianni et al. [96] discussed the involvement of ∆-9-desaturase in the process, but, as exposed by Ran et al. [95], the results were sometimes conflicting. Therefore, this is an area that should be studied in greater detail. Additionally, both supplemented organic minerals increased the UFA/SFA ratio. Ianni et al. [96] indicate that this result can be interpreted as a potential health benefit. The effects of increased ∑ ω6 in the selenium-supplemented group on human health should be evaluated since n6 PUFAs have generally been associated with a pro-inflammatory response, especially with an increased n6:n3 PUFA ratio [97]. In contrast, increased ∑ ω6 did not modify the ω6/ω3 ratio.

Although organic zinc supplementation did not alter milk production or DMI, there was a tendency for feed efficiency to be higher than the Control. Confirming the result found, Nayeri et al. [98], when studying Holstein cows from 28 days pre-partum to 250 days post-partum with supplementation of 75 mg of Zn/kg of DM, reported greater feed efficiency in the group supplemented with 40 mg in the form of zinc amino acid complex and 35 mg of zinc sulfate during lactation compared to the groups supplemented only with zinc sulfate or with 15.5 mg in the form of zinc amino acid complex and the remainder through zinc sulfate. Shakweer et al. [91] reported greater feed efficiency (milk production corrected for 4% fat/DMI) for Holstein cows, up to 182 days of DL, supplemented with 40 mg of zinc methionine/kg of DM compared to no supplementation and supplementation with 40 mg of zinc sulfate/kg of DM. In contrast, Oconitrillo et al. [11] reported no interference of organic zinc supplementation on feed efficiency despite having shown effects on DMI and treatment × time interaction on production. However, the comparison was between two groups of Holstein cows, with an average DL of 67 days, in which one received 76 mg of Zn/kg of DM in the form of zinc methionine and the other, an additional 20 mg of Zn/kg of DM from the same source, that is, two levels of supplementation in organic form were compared.

On the other hand, ruminal and digestibility parameters can help to understand the tendency of increased feed efficiency of the group supplemented with zinc. Wang et al. [22] showed that organic sources, such as zinc amino acid chelate and zinc proteinate, were more effective in increasing the total volatile fatty acids (VFAs), the concentration of bacteria, and the degradability of DM compared to zinc sulfate in an in vitro evaluation. In a complementary manner, Balabánová et al. [99] reported a tendency of the zinc methionine offer, compared to the same level of zinc oxide, to result in greater digestibility of crude protein, ether extract, crude fiber, non-nitrogen extract, and mineral matter in Holstein cows from 14 days pre-partum to 60 days post-partum. However, Chen et al. [23], when evaluating three supplementation levels (20, 40 and 60 mg of Zn/kg of DM) of zinc methionine for Holstein cows from 60 days pre-calving to calving, did not report any effects on apparent digestibility in the total tract (organic matter, NDF, ADF, crude protein and ether extract), but observed that increasing supplementation resulted in a linear increase in total VFA and microbial crude protein in the rumen. Given these discrepancies, this result should be the subject of future studies.

Organic selenium supplementation also showed a trend of greater feed efficiency than the Control, even without changes in milk production and DMI. Similarly, Li et al. [60] reported an increase in feed efficiency with supplementation of 0.1 and 0.3 mg Se/kg DM in the form of hydroxy-selenomethionine for Holstein cows in the first third of lactation when compared to the supplementation level of 0.5 and the control group (without selenium supplementation). In contrast, Sun et al. [78] showed that for Holstein cows in early lactation under the same supplementation level (0.3 mg Se/kg DM), replacing sodium selenite with hydroxy-selenomethionine did not change feed efficiency. However, efficiency was calculated differently (milk production corrected for 4% fat/DMI), and equal levels of selenium in the diet were compared, unlike the present study and Li et al. [60]. Additionally, Wang et al. [93] pointed out that supplementation of an organic form of selenium, selenium yeast, was able to alter digestibility parameters in Holstein cows. Supplementation of 0.15 and 0.3 mg of Se/kg of DM, compared to 0.45 and the control (0.07 mg of Se/kg of DM), increased the apparent digestibility in the total tract of dry matter, organic matter, crude protein, ether extract, NDF, and ADF.

Although the supplementation of minerals in organic form improved the health status of animals by stimulating immunity, anti-inflammatory indicators, antioxidant activity, reducing SCC (mammary gland health), and improving feed efficiency, it could not maintain superior lactation persistence. Lactation persistence can be defined as the ability to maintain milk production throughout lactation [100]. The lack of effect in this study may be related to the lactation period in which the cows used, that is, the end of lactation, when a decline in production naturally occurs [101]. Furthermore, the DL exceeded the ideal of 305 days [102].

From another perspective, the BCS of the group supplemented with organic zinc was higher on days 15 to 28 compared to the Control. The increase in feed efficiency of the Zinc group compared to the Control group discussed above is complementary to understanding the present result because it demonstrates greater efficiency in using ingested nutrients. However, studies with different strategies of zinc supplementation in organic forms for lactating cows reported no interference in the BCS [11,16,98,103]. It is emphasized that the difference in the BCS was subtle and possibly only detected by the greater precision of the automated milking robot system compared to human visual assessment. In addition, the BCS presented by the animals can be considered adequate for the end of lactation, considering that they are of the Jersey breed, and an increase in the BCS in this period would be undesirable.

Both supplemented groups had lower serum fructosamine concentrations. Fructosamine is formed by a sugar reaction, mainly glucose, with a protein (usually albumin) [104]. Thus, it indicates energy metabolism since its concentration is dependent on glucose. In an additive manner, the serum fructosamine concentration does not fluctuate due to acute variations in glucose, and it is constant throughout the day [105], being an indicator less subject to variations than glucose. It is known that zinc acts in the regulation of energy metabolism and participates in glucose homeostasis [106] and that selenium affects the expression of genes related to the mediation of metabolism and glucose transport [94]. Therefore, given the other results, reducing serum fructosamine levels can be interpreted as a better use of glucose. The proposed reference range for fructosamine in cattle (213.4 to 265.0 µmol/L) [105] reinforces this interpretation since the serum levels of the animals in the present study were above the reference and the decrease in values would mean an approximation of what is considered adequate.

Interestingly, supplementation with organic zinc and selenium reduced serum iron levels. Dresler et al. [107] reported that zinc supplementation in the form of zinc methionine could not influence serum iron concentration. However, the maximum supplementation level was 724 mg/cow/day, lower than that used in the present study. When studying humans, Donangelo et al. [108] stated that zinc and iron compete during intestinal absorption. They suggested the possibility that zinc supplementation (for young adult women with low iron stores) reduces the level of iron stores, indicated by a decrease in plasma ferritin concentration, which was accompanied by increased iron absorption. In contrast, it is known that the use of the transporter for which zinc and iron can compete, DMT1, is considered a minority pathway for zinc absorption [19]. It should be noted that the serum iron levels of the animals in the present study respected or were slightly above the range found by Noaman [109] for cows with six to nine months of gestation (268 ± 44 µg/dL). Regarding selenium, a study with humans reported that the increase in serum selenium concentration was related to the increase in serum iron concentration [110]. Given the above, it is evident that the interactions of zinc and selenium with iron are still poorly understood and need to be elucidated. Furthermore, the reduction in iron levels caused by supplementation should be further investigated since it can be interpreted differently: supplementation negatively affected iron levels (antagonism), or supplementation improved iron utilization, resulting in a decrease in circulating iron. The context of the results seems to indicate other interpretations.

**Figure 2:**
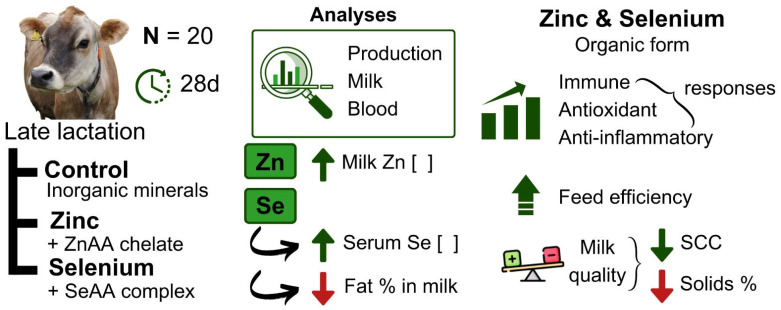
Schematic of the experiment and its main results using Jersey cows supplemented with zinc and selenium.

The findings were very interesting and met our main objectives, that is, the effects on improving cow health and increasing minerals in milk, seeking a food (milk) with nutraceutical power; since human consumption of zinc and selenium in food is desirable. However, it is important to make it clear to the reader that we considered the results preliminary, because this study had limitations in terms of the number of animals, in addition to having a short experimental period, without evaluating the effects after supplementation in the transition period. However, on the other hand, the number of variables analyzed with the effect of supplementation was considerable, showing how the intake of zinc and selenium can be beneficial to cows in the final third of lactation. The main results are presented in Figure 2 in a schematic form.

Despite the positive results for the cow, future studies will be necessary to verify the effects of these minerals in the long term, as well as in other phases of lactation; in order to understand whether the benefits to the cow’s health are maintained, or whether over time they may lead to intoxication of the animal with these organic sources. We do not understand the mechanisms involved in some metabolic, immunological and oxidative pathways, so new research with this focus is important. In addition, possible interactions with other nutrients can also be explored and thus help to explain the underlying mechanisms.

## 5. Conclusions

Supplementation of organic zinc and selenium in dairy cows at the end of lactation, in the form of chelated zinc amino acid and selenium amino acid complex, had positive effects on humoral immunity, antioxidant stimulation and reduction of lipid peroxidation, as well as demonstrated anti-inflammatory potential. Zinc and selenium supplementation modulated the phosphotransfer network, reducing CK activity and increasing AK and PK activity. Supplementation of both minerals implies in the bioavailability of serum iron, which reduces. The concentration of zinc in the milk of the group supplemented with organic zinc was higher, indicating greater mineral bioavailability. Supranutritional selenium intake reduced fat in cows’ milk, but selenium modulated the fatty acid profile in milk, with emphasis on the reduction of saturated fatty acids and an increase in the proportion of unsaturated fatty acids, combined with an increase in omega 6. Both minerals provided a reduction in the SCC count, improving milk quality.

## Figures and Tables

**Table 1:** Animal characteristics used to formulate the diets.

Variables	Information Pre-Experiment
Breed	Jersey
Animal type	Lactating cows
Age	43 months
Body weight	420 kg
Days in lactation	330
Days in gestation	190
Daily milk production	15 kg
Milk fat	4.50%
Protein in milk	3.26%

**Table 2:** Centesimal composition and minerals of interest levels in the experimental diets.

Composition of Diets Provided in Individual Feeders (kg DM ^2^/day)			
	Control	Zinc	Selenium
Corn silage	4.520	4.520	4.520
Tifton Hay 85	0.704	0.704	0.704
Concentrate			
Ground corn (2 mm)	2.420	2.420	2.420
Soybean meal (45% CP ^2^)	1.420	1.420	1.420
DDGs ^2^	0.790	0.790	0.790
Wheat bran	0.220	0.220	0.220
Soybean hulls	0.230	0.230	0.230
Bovimix Nucleus ^1^	0.180	0.180	0.180
Baking soda	0.096	0.096	0.096
Mycotoxin adsorbent	0.024	0.024	0.024
Chelated AA Zinc 22%	-	0.00220	-
Selenium complex AA ^2^ 0.5%	-	-	0.00215
**Level of Minerals of Interest in Total Diets (mg/kg DM)**			
	**Control**	**Zinc**	**Selenium**
Zinc	54.82	95.25	54.82
Selenium	00.30	00.30	01.19

Note 1: Nucleus Assurance Levels: Calcium (Min) 200 g/Kg. Calcium (Max) 285 g/Kg. Sulfur (Min) 15 g/Kg. Phosphorus (Min) 40 g/Kg. Magnesium (Min) 16 g/Kg. Sodium (Min) 76 g/Kg. Cobalt (Min) 11 mg/Kg. Copper (Min) 525 mg/Kg. Organic Chromium (Min) 29 mg/Kg. Iron (Min) 333 mg/Kg. Iodine (Min) 25 mg/Kg. Manganese (Min) 1430 mg/Kg. Selenium (Min) 16 mg/Kg. Zinc (Min) 2360 mg/Kg. Biotin (Min) 70 mg/Kg. Vitamin A (Min) 150,000 IU/Kg. Vitamin D3 (Min) 50,000 IU/Kg. Vitamin E (Min) 1000 IU/Kg. Monensin sodium 700 mg/Kg. Note 2: DM = dry matter; CP = crude protein; DDGS = distiller’s dried grains with soluble; AA = amino acid.

**Table 3:** Chemical composition of the basal diet.

Variables, %	Basal Diet
Dry matter (DM, %)	40.9
Mineral matter, % DM	7.20
Crude protein, % DM	16.2
Ether extract, % DM	2.80
Neutral detergent fiber—NDF, % DM	38.1
Acid detergent fiber—ADF, % DM	20.4

**Table 4:** Blood serum and milk levels of zinc and selenium.

Variable	Control	Zinc	Selenium	SEM	P: Treat
Zinc in serum, µg/g					
d 0 and 28	<1.80	<1.80	<1.80	-	-
Zinc in milk, µg/g					
d 28	3.76 ^b^	4.37 ^a^	3.72 ^b^	0.13	0.05
Selenium in serum, µg/g					
d 0	0.121	0.118	0.113	0.003	0.82
d 28	0.066 ^b^	<0.030 ^c^	0.087 ^a^	0.002	0.01
Selenium in milk, µg/g					
d 0 and 28	<0.030	<0.030	<0.030	-	-

Note: Different letters in the same row show statistical differences between groups (*p* ≤ 0.05).

**Table 5:** Hemogram and serum biochemistry blood supplemented with organic zinc and selenium.

Variable	Control	Zinc	Selenium	SEM	P: Treat	P: Day	P: Treat × Day
Hemogram							
Erythrocytes, ×10^6^/µL	6.22	6.10	6.24	0.04	0.95	0.98	0.97
Hemoglobin, mg/dL	11.2	11.2	11.1	0.14	0.94	0.95	0.92
Hematocrit, %	31.5	31.6	31.3	1.56	0.91	0.88	0.95
Platelets, ×10^3^/µL	294	284	319	12.8	0.23	0.65	0.18
Leukocytes, ×10^3^/µL	5.25 ^b^	6.08 ^a^	6.12 ^a^	0.29	0.09	0.13	0.36
Granulocytes, ×10^3^/µL	1.55	1.44	1.40	0.07	0.81	0.46	0.89
Monocytes, ×10^3^/µL	0.87	0.86	0.81	0.09	0.92	0.37	0.96
Lymphocytes, ×10^3^/µL					0.01	0.08	0.01
d 1	2.75	2.88	2.80	0.10			
d 14	3.27 ^b^	4.08 ^ab^	4.47 ^a^	0.12			
d 28	2.69 ^b^	3.46 ^a^	3.49 ^a^	0.11			
Serum biochemistry							
Cholinesterase, U/L					0.22	0.01	0.03
d 1	1325	1332	1242	41.0			
d 14	2134 ^a^	1717 ^b^	1823 ^b^	41.8			
d 28	1903 ^a^	1815 ^ab^	1645 ^b^	44.7			
Iron, µg/dL					0.01	0.01	0.01
d 1	217	203	196	5.81			
d 14	331 ^a^	260 ^b^	320 ^a^	7.46			
d 28	330 ^a^	259 ^b^	286 ^b^	7.24			
Globulin, g/dL					0.05	0.01	0.01
d 1	4.13	4.19	4.28	0.31			
d 14	4.27 ^b^	4.59 ^ab^	5.10 ^a^	0.29			
d 28	4.21	4.47	4.88	0.29			
Albumin, g/dL	3.90	3.67	3.60	0.21	0.78	0.35	0.84
Total protein, g/dL	8.15	8.20	8.59	0.36	0.53	0.41	0.37
Cholesterol, mg/dL	169	153	164	3.92	0.38	0.64	0.55
Fructosamine, µmol/L	349 ^a^	320 ^b^	322 ^b^	5.41	0.05	0.28	0.12
Urea, g/dL	49.0	52.0	48.0	1.50	0.92	0.81	0.85
Calcium, mg/dL	9.69	9.91	9.71	0.15	0.80	0.20	0.92
Phosphorus, mg/dL	8.80	8.09	8.24	0.19	0.72	0.34	0.51
Magnesium, mg/dL	2.78	2.57	2.62	0.09	0.83	0.17	0.87
C-reactive protein, mg/dL	29.2	29.0	29.1	0.02	0.97	0.99	0.98
Ferritin, µg/L	484	486	494	4.06	0.84	0.42	0.75

Note: Different letters in the same row show statistical differences between groups (*p* ≤ 0.05) and trends (*p* > 0.05 and *p* ≤ 0.10).

**Table 6:** Biochemistry of energy metabolism in blood serum of Jersey cows supplemented with zinc and organic selenium.

Variable	Control	Zinc	Selenium	SEM	P: Treat	P: Day	P: Treat × Day
Creatine kinase, U/L					0.02	0.01	0.01
d 1	251	253	235	8.32			
d 14	167 ^a^	113 ^b^	162 ^a^	7.10			
d 28	242 ^a^	167 ^b^	182 ^b^	7.02			
Adenylate kinase, nmol ATP/min/mg protein					0.01	0.01	0.01
d 1	11.3	10.9	11.1	0.84			
d 14	11.7 ^b^	14.7 ^a^	14.6 ^a^	0.96			
d 28	11.0 ^b^	16.13 ^a^	15.0 ^a^	0.88			
Pyruvate kinase, nmol pyruvate/min/mg protein					0.01	0.01	0.01
d 1	79.1	78.4	79.6	1.55			
d 14	81.5 ^b^	90.2 ^a^	89.7 ^a^	1.82			
d 28	79.2 ^b^	90.1 ^a^	80.5 ^ab^	1.84			

Note: Different letters in the same row show statistical differences between groups (*p* ≤ 0.05).

**Table 7:** Oxidative status in serum of Jersey cows supplemented with organic zinc and selenium.

Variable	Control	Zinc	Selenium	SEM	P: Treat	P: Day	P: Treat × Day
ROS, % of fluorescence intensity					0.01	0.01	0.01
d1	184	175	185	6.84			
d14	232 ^a^	178 ^c^	199 ^b^	7.01			
d28	210 ^a^	147 ^b^	160 ^b^	6.67			
TBARS, nM of MDA/mL					0.18	0.02	0.05
d1	10.6	10.3	10.1	0.06			
d14	12.2	10.8	12.1	0.06			
d28	12.5 ^a^	10.7 ^b^	9.62^c^	0.05			
MPO, μMq/mg/30 min					0.01	0.01	0.01
d1	2.33	2.86	2.28	0.08			
d14	2.29	1.86	1.91	0.07			
d28	2.37 ^a^	1.62 ^b^	1.30 ^b^	0.07			
PSH, µmol/L	4.31	4.11	3.74	0.14	0.12	0.25	0.14
NPSH, µmol/L	1.06	1.15	1.14	0.06	0.85	0.92	0.65
Vitamin C, µg/dL	119	103	107	5.51	0.15	0.35	0.27

Note 1: Different letters in the same line show statistical differences between groups (*p* ≤ 0.05). Note 2: ROS = reactive oxygen species; TBARS = thiobarbituric acid-reactive substances; MPO = myeloperoxidase; PSH = total thiols; NPSH = nonprotein thiols.

**Table 8:** Milk composition and quality of cows supplemented with organic zinc and selenium.

Variable	Control	Zinc	Selenium	SEM	P: Treat	P: Day	P: Treat × Day
Total protein, %	4.00	4.11	4.10	0.31	0.25	0.21	0.45
Lactose, %	4.51	4.57	4.62	0.14	0.44	0.75	0.51
Fat, %					0.03	0.01	0.05
d 1	4.57	4.42	4.32	0.52			
d 14	4.18 ^a^	4.19 ^a^	3.59 ^b^	0.21			
d 28	5.68 ^a^	5.23 ^ab^	4.76 ^b^	0.22			
Total solids, %					0.39	0.05	0.08
d 1	14.2	13.9	13.8	0.37			
d 14	13.7 ^ab^	14.2 ^a^	13.1 ^b^	0.35			
d 28	15.4 ^a^	14.5 ^b^	14.7 ^b^	0.35			
SCC, ×10^3^/mL					0.01	0.01	0.01
d 1	129	109	132	14.3			
d 14	160 ^a^	94.6 ^b^	130 ^ab^	15.0			
d 28	242 ^a^	61.5 ^c^	180 ^b^	12.7			
MUN, %	22.1 ^a^	22.4 ^a^	19.9 ^b^	0.75	0.05	0.01	0.12

Note 1: Different letters in the same row show statistical differences between groups (*p* ≤ 0.05) and trends (*p* > 0.05 and *p* ≤ 0.10). Note 2: SCC = somatic cell count; MUN = milk urea nitrogen.

**Table 9:** Fatty acid profile in milk of cows supplemented with organic zinc and selenium.

Fatty Acid, %	Control	Zinc	Selenium	SEM	*p*-Value
C6:0 (Caproic)	0.42 ^a^	0.39 ^a^	0.26 ^b^	0.026	0.05
C8:0 (Caprylic)	0.59	0.56	0.46	0.023	0.16
C10:0 (Capric)	2.07	2.00	1.62	0.076	0.24
C11:0 (Undecanoic)	0.18	0.18	0.15	0.006	0.79
C12:0 (Lauric)	3.02 ^a^	2.93 ^a^	2.39 ^b^	0.102	0.05
C13:0 (Tridecanoic)	0.12	0.11	0.10	0.003	0.97
C14:0 (Myristic)	12.0 ^a^	12.1 ^a^	10.4 ^b^	0.296	0.01
C14:1 (Myristoleic)	0.70	0.69	0.64	0.027	0.11
C15:0 (Pentadecanoic)	1.02	1.00	1.01	0.023	0.98
C16:0 (Palmitic)	40.6 ^a^	38.9 ^b^	38.8 ^b^	0.464	0.08
C16:1 (Palmitoleic)	1.11	1.19	1.27	0.100	0.29
C17:0 (Heptadecanoic)	0.46	0.46	0.50	0.011	0.65
C17:1 (cis-10-Heptadecenoic)	0.15	0.15	0.17	0.006	0.92
C18:0 (Stearic)	14.27	14.77	15.20	0.235	0.30
C18:1 n9t (Elaidic)	1.15 ^b^	1.34 ^ab^	1.48 ^a^	0.073	0.02
C18:1 n9c (Oleic)	19.1 ^b^	20.0 ^b^	22.2 ^a^	0.461	0.01
C18:2 n6c (Linoleic)	1.97	2.11	2.23	0.091	0.25
C20:0 (Arachidic)	0.19	0.20	0.20	0.003	0.95
C18:3 n6 (γ- Linolenic)	0.03	0.03	0.03	0.001	0.99
C20:1 n9 (cis-11-Eicosenoic)	0.05	0.05	0.06	0.002	0.98
C18:3 n3 (α-Linolenic)	0.14	0.14	0.14	0.005	0.97
C21:0 (Henicosanoic)	0.21	0.23	0.22	0.008	0.95
C20:2 (cis-11,14-Eicosadienoic)	0.04	0.05	0.04	0.001	0.96
C22:0 (Behenic)	0.06	0.06	0.06	0.002	0.99
C20:3 n6 (cis-8,11,14-Eicosatrienoic)	0.06	0.07	0.07	0.003	0.98
C20:4 n6 (Arachidonic)	0.06	0.05	0.05	0.001	0.98
C24:0 (Lignoceric)	0.05	0.05	0.06	0.002	0.97
C20:5 n3 (cis-5,8,11,14,17-Eicosapentaenoic)	0.01	0.02	0.02	0.001	0.98
C24:1 n9 (Nervonic)	0.01	0.01	0.01	0.001	0.99
C22:6 n3 (cis-4,7,10,13,16,19-Docosahexaenoic)	0.00	0.00	0.01	0.000	0.98
∑ Saturated fatty acids (SFA)	75.3 ^a^	74.0 ^a^	71.5 ^b^	0.572	0.01
∑ Unsaturated fatty acids (UFA)	24.6 ^b^	25.9 ^b^	28.4 ^a^	0.572	0.001
∑ Monounsaturated fatty acids (MUFA)	22.3^b^	23.4 ^ab^	25.8 ^a^	0.516	0.03
∑ Polyunsaturated fatty acids (PUFA)	2.31 ^b^	2.46 ^ab^	2.58 ^a^	0.096	0.06
UFA/SFA	0.32 ^c^	0.35 ^b^	0.39 ^a^	0.011	0.01
∑ ω6	2.11 ^b^	2.26 ^ab^	2.38 ^a^	0.091	0.04
∑ ω3	0.15	0.16	0.16	0.005	0.56
ω6/ω3	13.73	14.36	14.46	0.306	0.23

Note: Different letters in the same row show statistical differences between groups (*p* ≤ 0.05) and trends (*p* > 0.05 and *p* ≤ 0.10). Note 2: FCM = 4% Fat-Corrected Milk.

**Table 10:** Productive performance of cows supplemented with organic zinc and selenium.

Variable	Control	Zinc	Selenium	SEM	P: Treat
Milk production, kg					
d 1–14	13.6	13.9	13.7	0.15	0.97
d 15–28	11.8	11.9	12.1	0.12	0.91
d 1–28	12.7	12.9	12.9	0.14	0.93
FCM d 1–28, kg	14.5	14.0	12.6	0.87	0.16
Dry matter intake, kg					
d 1–14	11.7	11.6	11.5	0.09	0.96
d 15–28	11.7	10.9	11.0	0.09	0.25
d 1–28	11.7	11.2	11.2	0.08	0.71
Feed efficiency, kg/kg					
d 1–14	1.16	1.19	1.19	0.02	0.58
d 15–28	1.00 ^b^	1.10 ^a^	1.10 ^a^	0.02	0.07
d 1–28	1.08	1.15	1.15	0.02	0.13
Lactation persistence, %					
d 1–28	77.4	77.1	76.0	1.89	0.92
Body condition score					
d 1–14	3.29	3.30	3.43	0.12	0.76
d 15–28	3.35 ^b^	3.58 ^a^	3.42 ^b^	0.09	0.05

Note 1: Different letters on the same line show statistical differences between groups (*p* ≤ 0.05) and trends (*p* > 0.05 and *p* ≤ 0.10). Note 2: FCM = 4% Fat-Corrected Milk.

## Data Availability

Data and materials can be made available upon request.

## Supplementary Materials

The following supporting information can be downloaded at: https://www.mdpi.com/article/10.3390/ani15040499/s1, Table S1. Chemical composition of silage, hay, concentrate, and pelleted concentrate; Table S2. Concentrates and TMR levels of zinc and selenium; Table S3. Fatty acid profile of the basal diet; Table S4. Expected and effectively supplied zinc and selenium levels in mg/animal/day.
