# Supplementary material for: Organic Zinc and Selenium Supplementation of Late Lactation Dairy Cows: Effects on Milk and Serum Minerals Bioavailability, Animal Health and Milk Quality
Source: Animals (Basel). 2025 Feb 10;15(4):499. doi: 10.3390/ani15040499 (PMC11852322; doi:10.3390/ani15040499)
Supplement: Supplementary file 1 [file animals-15-00499-s001.zip › animals-3433222-supplementary.pdf]

animals

MDPI

SUPPLEMENTARY information

Table S1. Chemical composition of silage, hay, concentrate, and pelleted concentrate

Variables, %	Silage	Hay	Concentrate	Pelleted concentrate
Dry matter (%)	23.01	84.11	87.29	86.72
Mineral matter, % DM	05.62	06.91	06.56	05.93
Crude protein, % DM	09.43	11.76	25.11	21.06
Ether extract, % DM	02.66	01.58	03.17	02.77
NDF, % DM	54.02	74.10	20.74	21.28
ADF, % DM	30.24	35.78	09.41	08.98

Note: DM = dry matter; NDF = neutral detergent fiber; ADF = acid detergent fiber.

Table S2. Concentrates and TMR levels of zinc and selenium

Variables	Control	Zinc	Selenium
Levels reached in the concentrate, mg/kg DM			
Zinc	80.92	151.64	80.92
Selenium	0.52	0.52	1.76
Levels achieved in the total diet, mg/kg DM			
Zinc	43.89	82.24	43.89
Selenium	0.28	0.28	0.95
Expected levels in total diet based on formulation, mg/kg DM			
Zinc	54.82	95.25	54.82
Selenium	0.30	0.30	1.19
Difference between achieved and expected levels in the total diet, mg/kg DM			
Zinc	-10.93	-13.01	-10.93
Selenium	-0.02	-0.02	-0.24
Expected levels of organic supplementation in the total diet, mg/kg DM			
Zinc	-	40.43	-
Selenium	-	-	0.89
Levels achieved in organic form in the total diet, mg/kg DM			
Zinc	-	38.35	-
Selenium	-	-	0.67
Difference between achieved and expected levels in organic form, mg/kg DM			
Zinc	-	–1.58	-
Selenium	-	-	–0.22

Table S3. Fatty acid profile of the basal diet

**Fatty acids, %**	**Basal diet**
C10:0 (Capric)	00.06
C12:0 (Lauric)	00.16
C13:0 (Tridecanoic)	00.10
C14:0 (Myristic)	00.54
C14:1 (Myristoleic)	00.06
C15:0 (Pentadecanoic)	00.13
C16:0 (Palmitic)	22.49
C16:1 (Palmitoleic)	00.28
C17:0 (Heptadecanoic)	00.23
C17:1 (cis-10-Heptadecenoic)	00.06
C18:0 (Stearic)	04.61
C18:1 n9t (Elaidic)	00.25
C18:1 n9c (Oleic)	24.57
C18:2 n6c (Linoleic)	39.61
C20:0 (Arachidic)	00.65
C20:1 n9 (cis-11-Eicosenoic)	00.22
C18:3 n3 (α-Linolenic)	04.66
C22:0 (Behenic)	00.56
C20:4 n6 (Arachidonic)	00.14
C24:0 (Lignoceric)	00.59
C24:1 n9 (Nervonic)	00.09
∑ Saturated fatty acids (SFA)	30.09
∑ Unsaturated fatty acids (UFA)	69.91
∑ Monounsaturated fatty acids (MUFA)	25.50
∑ Polyunsaturated fatty acids (PUFA)	44.40
UFA/SFA	02.32
∑ ω6	39.75
∑ ω3	04.66
ω6/ω3	08.62

Table S4. Expected and effectively supplied zinc and selenium levels in mg/animal/day

**Variable**	**Control**	**Zinc**	**Selenium**
**Zinc, mg/animal/day**			
Requirement calculated based on formulation	602.00	602.00	602.00
Expected supply levels	662.23	1150.62	662.23
Supply levels reached	513.51	921.09	491.57
Difference between achieved and expected levels	–148.72	–229.53	–170.66
Recalculated requirement	546.50	538.00	538.00
Difference between achieved level and recalculated requirement	–32.99	383.09	–46.43

Expected level of organic supplementation	-	488.39	-
Level reached in organic form	-	429.52	-
Difference between achieved and expected organic level	-	–58.87	-
**Selenium, mg/animal/day**			
Expected supply levels	3.62	3.62	14.38
Levels reached	3.28	3.14	10.64
Difference between achieved and expected levels	–0.34	–0.48	–3.74
Expected level of organic supplementation	-	-	10.75
Level reached in organic form	-	-	7.50
Difference between achieved and expected organic level	-	-	–3.25
